# The effect of orthodontic bracket base shape on shear bond strength to human enamel, an *in vitro* study

**DOI:** 10.2340/biid.v11.40585

**Published:** 2024-05-07

**Authors:** Ziana Esmail, William Wiltshire, Fabio H. S. L. Pinheiro, Carolina M. Frota, Rodrigo França

**Affiliations:** a Department of Preventative Dental Science, Division of Orthodontics, University of Manitoba, Winnipeg, Manitoba, Canada; b Department of Restorative Dentistry, University of Manitoba, Winnipeg, Manitoba, Canada

**Keywords:** shear bond strength, shaped brackets, orthodontic bracket base shape, adhesive remnant index

## Abstract

The purpose of this study was to evaluate the *in vitro* effect of orthodontic bracket base shape on shear bond strength (SBS) to human enamel and assess the nature of debonding fractures using the Adhesive Remnant Index (ARI). Orthodontic brackets with different-shaped bases (flower, heart, rectangle) were bonded to 120 extracted human third molars. Shear bond strength was measured using a Servohydraulic Test System at 24 h and 2 months after bonding. Adhesive Remnant Index scores were evaluated under 10x magnification to assess the amount of resin left on the tooth. The control bracket (rectangular base shape) had the highest mean SBS (26.8 ± 8.2 megapascals [MPa]), and significantly differed from the flower (17.2 ± 4.4 MPa) and heart (18.9 ± 3.5 MPa) base shapes (*p* < 0.001). The mean SBS between debonding times at 24 h (21.5 ± 7.4 MPa) and 2 months (20.4 ± 6.7 MPa) were not statistically significant *(p* > 0.05). Analysis of ARI scores showed a significant difference between flower-24 h versus heart-2 months (*p* = 0.039), flower-24 h versus heart-24 h (*p* = 0.004), and control-2 months versus heart-24 h (*p* = 0.015). Bracket base shape influenced SBS, with the rectangular base shape having a higher mean SBS compared to flower and heart base shapes. Variations in ARI scores occurred based on bracket shape and were of a mixed adhesive-cohesive nature. All bracket shapes had bond strengths above the clinically acceptable range of 6–8 MPa, and may thus provide adequate SBS in a clinical situation.

## Introduction

The bond strength of an orthodontic bracket needs to be high enough to withstand normal masticatory and orthodontic forces. Concomitantly, it also needs to be weak enough to allow easy removal without damaging the enamel [1]. During mastication, the average force exerted on a bracket is between 40 and 120 N; therefore, the force required to debond a bracket should be greater than 120 N [2, 3].

Bond failures are often a combination of both adhesive and cohesive failure [4]. Failure at the interface between the bonding material and the bracket is most desirable, as it minimises the risk of damage to the enamel [5]. The Adhesive Remnant Index (ARI) allows one to evaluate the type of bond failure and measure the amount of resin remaining on the tooth after bracket removal [3, 6].

The increasing popularity of different bracket bases reflects a growing demand for unique and personalised orthodontic accessories among young consumers. These bracket bases, which deviate from the traditional rectangular shape, offer a range of eye-catching designs including hearts, soccer balls, footballs, stars, flowers, and diamonds. This diverse selection caters to the individual preferences and personalities of orthodontic patients, allowing them to express themselves through their braces in a way that was previously unavailable. As a result, the industry has successfully tapped into a niche market, providing a creative and customisable option for those seeking to make a statement with their dental appliances. However, as they are bonded directly to the enamel surface, these different formats may affect the bond strength and fracture pattern. To date, the two predominant studies that have investigated the effect of base shapes on shear bond strength (SBS) to enamel present contradicting results. A study by Pham et al. [7] demonstrated that the bracket base shape has an effect on the SBS to bovine enamel. There was a superior bond strength for rectangular (control), flower, and football shapes, as these geometrical shapes may allow for more even stress distribution throughout the bracket base. The diamond and heart shapes had a lower SBS. Another study by Patel et al. [8] evaluated the effect of orthodontic bracket base shape on SBS to human enamel. Their study illustrated that orthodontic bracket base shape has no effect on SBS, nor does it affect the mode of fracture pattern [8]. As these two noteworthy studies have inconsistent results, it is beneficial to investigate this further.

Therefore, the purpose of the present *in vitro* study was to evaluate the effect of orthodontic bracket base shape on SBS to human enamel, at two time points, as well as the nature of debonding fractures using the ARI. The study evaluated one shape with superior bond strength (flower) compared to one shape with inferior bond strength (heart) as reported by Pham et al. [7] The null hypothesis was that there were no statistically significant differences for SBS and ARI.

## Materials and methods

The study obtained ethics approval from the Research Ethics Board of the institution where it was conducted.

### Materials

The teeth were brushed with distilled water and cut at the cementoenamel junction using a Torit model trimmer. The roots were discarded, and the crowns were embedded in a self-curing acrylic with a polyvinyl chloride (PVC) cylindrical mould. The control brackets (Master Series®, American Orthodontics, Sheboygan, WI, USA) had a traditional semi-rectangular base shape. The average bracket base surface area of five randomly selected rectangular brackets, measured using Image-Pro software (Media Cybernetics, Rockville, MD, USA), was 10.42 mm^2^. The flower and heart-shaped brackets were provided by WildSmiles® (Omaha, NE, USA), and their base surface area was informed by the manufacturer as illustrated in Table 1. All brackets were upper right central incisors with a 0.018-inch slot size.

**Table 1:** Summary of the brackets used for bonding.

Group	Number of teeth	Bracket	Surface Area (mm^2^)	Image
1	40	Standard Bracket(Master Series®, American Orthodontics)	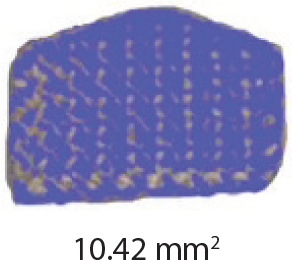	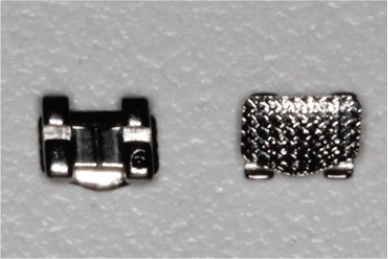
2	40	Flower shaped Bracket(WildSmiles®)	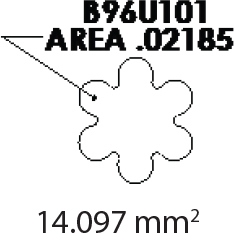	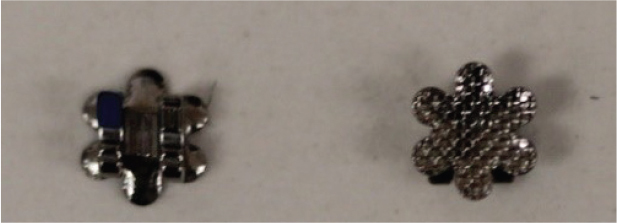
3	40	Heart shaped Bracket(WildSmiles®)	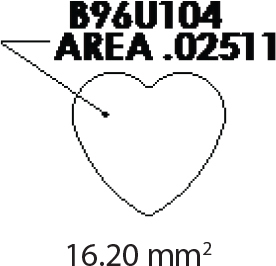	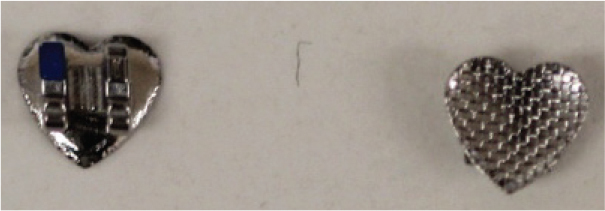

### Bonding procedures

The sample was divided into three equal groups: (1) the control group with traditional rectangular brackets, (2) flower-shaped WildSmiles® brackets, and (3) heart-shaped WildSmiles® brackets.

Prior to bonding, the labial surface of the teeth was cleaned with a non-fluoridated, non-flavoured prophy paste (Ortho Technology, Tampa Bay, FL), and a rubber cup for 15 s, then rinsed and dried for 20 and 10 s, respectively.

The centre of the teeth was etched for 15 s using Etch-Rite™ 38% phosphoric acid (Pulpdent, Oakland, MA, USA), rinsed with water spray for 20 s and air-dried for 20 s until a frosty appearance was present on the enamel surface. Transbond™ XT Light Cure Adhesive Primer (3M Unitek, Monravia, CA, USA) was applied to the etched enamel surface with a microbrush, gently air-thinned, and light cured for 6 s using the Ortholux™ Luminous curing light (3M Unitek, Monravia, CA, USA). The bracket mesh pad was coated with a uniform amount of Transbond™ XT Light Cure Adhesive Paste and pressed against the teeth with a bracket holder. To ensure uniform and complete seating of the bracket to the tooth, a 500 g weight was used. The application of a fixed load to the bracket produces more consistent results [9]. Any excess resin was removed using a periodontal probe. The teeth were light-cured for 6 s mesial and 6 s distal to the bracket using the Ortholux™ Luminous curing light as per the manufacturer’s guidelines. After initial bonding, the teeth were stored in glass containers filled with distilled water and in an incubator (Precision Scientific, Thelco Model Z) at 37°C to simulate oral conditions.

### Shear Bond Strength procedure

Each group was divided into two-time points and underwent shear bond strength (SBS) tests as follows: 24 h after initial bonding to evaluate early bond strength (T1), and 2 months after initial bonding to evaluate delayed bond strength after complete water sorption equilibration and bond maturation (T2).

The teeth were mounted into the Bencor Multi-T testing apparatus (Danville Engineering, San Ramon, CA, USA) with a knife-edge shearing blade positioned at the enamel-resin interface. The Bencor Multi-T testing apparatus was placed in the MTS Landmark® Servohydraulic Test System (Eden Prairie, Minnesota, USA), which was used to record the SBS with a crosshead speed of 0.5 mm/min using a 1.3 kN (300 lb) load cell. The testing machine was linked to a computer, where the measurements were recorded in Newtons (N). Shear bond strength in megapascals (MPa) was calculated using the formula: 1 MPa = 1 N/mm^2^, where mm^2^ refers to the bracket base surface area.

### Evaluation of Adhesive Remnant Index

Following the debonding of the bracket, an ARI score was evaluated visually under a Leica EZ4 Stereo microscope at 10x magnification. A modified ARI score [10] was used to quantify the amount of resin remaining on the tooth using the following scale: 1 = all the resin composite remaining on the tooth (100%) with the impression of the bracket base, 2 ≥ 90% composite remaining on the tooth, 3 = between 10 and 90% composite remaining on the tooth, 4 ≤ 10% composite remaining on the tooth, and 5 = no composite remaining on the tooth. To ensure reliability, 30% of the sample was randomly chosen to undergo intra- and inter-rater reliability tests.

### Distortion analyses

After debonding, the brackets were collected. These brackets were carefully cleaned without causing additional distortion. The evaluation was performed using a Leica EZ4 stereo microscope at 10x magnification. Measurements were taken to quantify any distortions or changes in bracket geometry. The post-debonding data were compared against the specifications of new brackets and against pre-debonding data.

### Statistical analysis

A two-way Analysis of Variance (ANOVA) and post-hoc Sidak test were used to compare the mean SBS values among the different groups. A Kruskal-Wallis test and post-hoc Dunn-Bonferroni test were used to compare differences in ARI scores across bracket type and debond time. The weighted kappa statistic was used to calculate inter- and intra-rater agreements. All statistical analyses were performed using IBM SPSS Statistics for Windows, Version 27.0 (IBM Corp., Armonk, NY), with the significance level set at *p* < 0.05.

**Figure 1:**
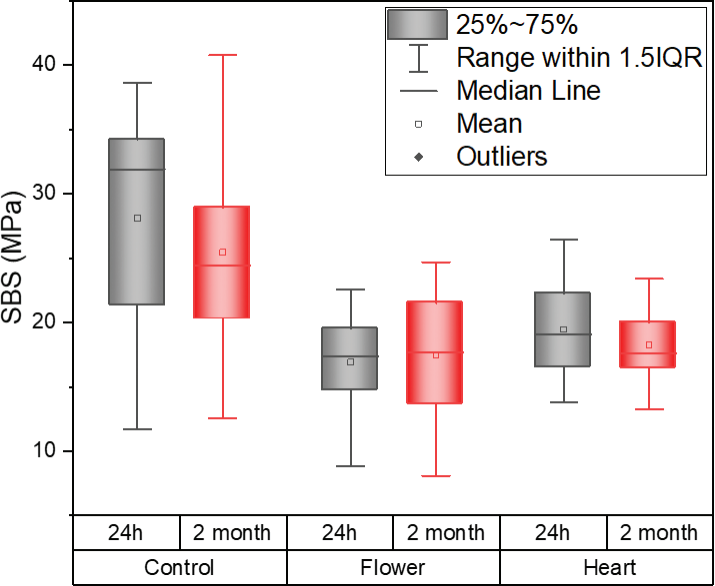
Shear bond strength results of different brackets bases according to the storage time.

## Results

### Shear bond strength

The descriptive statistics for SBS of all groups are listed in Table 2.

**Table 2:** Descriptive statistics of shear bond strength by bracket type and debond time.

Bracket Type – Debond Time	*N*	Mean (MPa)	Standard Deviation	Minimum (MPa)	Maximum (MPa)	Min-Max Range (MPa)	Coefficient of Variation
Control – 24 h	20	28.1	8.6	11.73	38.65	26.92	30.44 %
Flower – 24 h	20	16.9	3.5	8.87	22.56	13.69	20.87 %
Heart – 24 h	20	19.4	3.8	13.80	26.45	12.65	19.66 %
Control – 2 months	20	25.4	7.8	12.54	40.78	28.24	30.58 %
Flower – 2 months	20	17.4	5.3	8.07	24.67	16.60	30.39 %
Heart – 2 months	20	18.3	3.1	13.27	23.39	10.12	17.11 %

The two-way ANOVA revealed a significant difference in the mean SBS between different bracket types (*F* = 31.695, *p* < 0.001). A post-hoc *Sidak test* was done to determine where the significant difference in SBS existed between bracket types, as shown in Table 3. The control bracket with a rectangular base shape had the highest mean SBS (26.8 ± 8.2 MPa), and significantly differed from flower (17.2 ± 4.5 MPa) and heart (18.9 ± 3.5 MPa) base shapes (*p* < 0.001). The mean SBS of WildSmiles-shaped brackets, flower and heart shaped brackets, did not differ significantly from each other *(p* > 0.05).

**Table 3:** Pairwise comparisons of shear bond strength between bracket types.

Bracket Shape (*N* = 40)		*p*-value
Control Mean SBS = 26.8 ± 8.2 MPa	**Flower**	0.000*
	**Heart**	0.000*
Flower Mean SBS = 17.2 ± 4.5 MPa	**Control**	0.000*
	**Heart**	0.482
Heart Mean SBS = 18.9 ± 3.5 MPa	**Control**	0.000*
	**Flower**	0.482

* *p* < 0.05 statistically significant.

Mean SBS between debond times at 24 h (21.5 ± 7.5 MPa) and 2 months (20.4 ± 6.7 MPa) was not considered statistically significant with a probability value of 0.291 (*F* = 1.124, *p* > 0.05). The interaction of bracket type versus debond time was also not considered statistically significant with a probability value of 0.467 (*F* = 0.737, *p* > 0.05).

### Adhesive Remnant Index

The frequency distribution of modified ARI scores for all bracket types at each debond time is illustrated in Table 4. Amongst all groups, ARI score 1 was the least frequent at 1/120 (0.8%), and ARI score 3 was the most frequent at 46/120 (38.3%).

**Table 4:** Frequency and percentage of Adhesive Remnant Index scores.

Group	ARI Score					Total	Median ARI Score	Interquartile Range
	1	2	3	4	5			
**Control – 24 h ^abc^**	0 (0%)	4 (20%)	4 (20%)	**8 (40%)**	4 (20%)	20	4.00	1
**Flower – 24 h ^a^**	0 (0%)	3 (15%)	**12 (60%)**	5 (25%)	0 (0%)	20	3.00	1
**Heart – 24 h ^c^**	1 (5%)	0 (0%)	2 (10%)	**10 (50%)**	7 (35%)	20	4.00	1
**Control – 2 months ^ab^**	0 (0%)	5 (25%)	**9 (45%)**	3 (15%)	3 (15%)	20	3.00	2
**Flower – 2 months ^abc^**	0 (0%)	1 (5%)	**12 (60%)**	5 (25%)	2 (10%)	20	3.00	1
**Heart – 2 months ^bc^**	0 (0%)	0 (0%)	**7 (35%)**	6 (30%)	**7 (35%)**	20	4.00	2
**TOTAL**	**1 (0.8%)**	**13 (10.8%)**	**46 (38.3%)**	**37 (30.8%)**	**23 (19.2%)**	**120**		

a-c Groups not sharing any letter are significantly different (*p* < 0.05).

The *Kruskal-Wallis test* showed a significant difference in mean ranks of ARI scores across bracket type and debond time (X^2^ = 21.373, *p* = 0.001). Pairwise comparisons using the post-hoc *Dunn-Bonferroni test* demonstrated significant differences in ARI scores between Flower–24 h versus Heart–2 months (*p* = 0.039), Flower–24 h versus Heart–24 h (*p* = 0.004), and Control–2 months versus Heart –24 h (*p* = 0.015). Median and interquartile range (IQR) values can be found in Table 4.

Intra-rater reliability test found that 94% of the ARI scores were the same as the initial evaluation with a *weighted kappa statistic* of 0.942. This is considered almost perfect agreement when kappa > 0.9 [11]. For inter-rater reliability testing, 88.9% of the ARI scores were the same between the principal investigator and the third party. The *weighted kappa statistic* was 0.824, which suggests strong agreement approaching near-perfect agreement [11].

### Bracket base distortion

Upon examination of the brackets after bond strength testing, the shaped brackets demonstrated bracket base distortion. The flower-shaped WildSmiles® bracket exhibited the greatest number of distorted bracket bases with 19/20 (95%) and 18/20 (90%) at the 24-h and 2-month debond times, respectively. The heart-shaped WildSmiles® bracket displayed much less distortion, with only 1/20 (5%) brackets distorted at 24 h, and 6/20 (30%) brackets distorted at 2 months. The rectangular-shaped control bracket did not exhibit any visible distortion after debonding.

## Discussion

Based on the findings of this study, the null hypothesis was rejected, indicating a statistically significant difference in mean SBS values among different bracket shapes. Specifically, the rectangular-shaped control bracket demonstrated a higher mean SBS compared to the flower and heart shapes. It is worth noting, however, that all SBS values for each bracket type exceeded the clinically acceptable range of 6–8 MPa. Therefore, it is reasonable to speculate that shaped bracket bases would perform satisfactorily in a clinical setting. Additionally, the study found no significant difference between debond times at 24 h and 2 months. These results provide valuable insights into the performance of different bracket shapes and their potential clinical implications.

The investigation findings indicate that the SBS of brackets is not solely dependent on its base surface area. The rectangular base (10.42 mm^2^) had a smaller surface area but a greater mean SBS, whereas the flower (14.10 mm^2^) and heart (16.20 mm^2^) shaped brackets had a larger surface area and a lower mean SBS. This is in agreement with previous studies that demonstrated that bond strength is independent of surface area, as long as the bracket base area is at least 6.82 mm^2^ or larger [2, 12, 13].

In orthodontic practice, an excessively high bond strength can increase the likelihood of enamel fractures during debonding. Studies have suggested that bond strengths exceeding certain thresholds may pose a risk to enamel integrity, with some advocating for bond strengths not surpassing 10 MPa, which is close to the tensile strength of enamel. Therefore, while high bond strength is desirable for effective orthodontic treatment, it is equally crucial to balance it with the preservation of enamel health.

Considering these factors, the rectangular base shape, despite showing superior bond strength in this study, may raise concerns about potential enamel damage risks during bracket removal. Practitioners must carefully assess the trade-off between bond strength and enamel safety when selecting bracket types for orthodontic treatments. Balancing the need for adequate bond strength with the preservation of enamel integrity is essential for ensuring successful and safe orthodontic outcomes in clinical practice.

Differences in bond strength testing protocols may challenge the comparison of results across various studies [14]. In this study, the shear force was applied at the enamel-resin interface. The studies that previously investigated WildSmiles® shaped brackets [7, 8], placed the shear debonding force at the bracket ligature groove. Klocke and Kahl-Nieke [15] reported a decrease in SBS by 49.3% when the force application was moved from the bracket-enamel interface to the ligature groove. As the distance between the applied force and the enamel surface increases, a moment of force is introduced, and there are greater components of tensile, compressive, and peel stress, rather than pure shear stress [7, 15].

Although higher SBS values may reduce undesirable bond failures and emergency appointments, there might be a greater risk of enamel damage when the brackets are removed at the end of treatment. Retief [16] reported enamel fractures in bond strengths as low as 9.7 MPa, whereas other authors did not report any enamel fractures until the bond strength exceeded 16 MPa [5, 17]. Nkenke et al. [18] noted that bond strength should not surpass 10 MPa, which is the tensile strength of enamel. The mean SBS values obtained in this study exceed 6–8 MPa, reported in the literature as a clinically acceptable standard for *in vitro* SBS testing [1, 6, 19, 20]. Because higher bond strength values can be associated with possible iatrogenic damage to the enamel surface, one should be cautious when debonding any brackets which exceed these values [8]. However, the direct implications to clinical practice are unknown, since this study did not assess possible damages to the enamel surface as it focussed on other debonding outcomes.

The analysis of ARI indicates that most bond failures were a combination of adhesive-cohesive, with ARI scores 3 and 4 being the most common at 38.3% and 30.8% respectively. The heart-shaped bracket had a higher percentage of resin remaining on the bracket base, with 35.0% receiving an ARI score of 5, suggesting a greater proportion of adhesive failures at the enamel-adhesive interface. This increases the risk of iatrogenic enamel fracture upon debond [5, 18, 21]. On the other hand, failure at the bracket-adhesive interface results in most residual adhesive remaining on the tooth surface, requiring either rotary or hand instrumentation for removal.

The flower-shaped bracket exhibited the greatest number of distorted bracket bases (90–95%) and the heart-shaped bracket had a few distorted brackets (5–30%). Because the heart-shaped bracket exhibited much less base distortion, it had higher ARI scores and more adhesive-cohesive bond failures. Conversely, the flower-shaped bracket displayed a greater amount of bracket base distortion, thus had lower ARI scores, and more adhesive-cohesive bond failures. The heart-shaped bracket has a converging sharp tip at the incisal base extension. Sharp edges on a bracket base can lead to stress concentration [12], subsequent crack formation, and eventually bond failure [22, 23]. This may explain why variations in ARI scores occurred based on bracket shape. In addition, bracket distortion may be a product of the knife-edge shear testing blade and may not necessarily be relevant to the clinical situation. These findings indicate that the level of base distortion in different bracket shapes can significantly impact the adhesive performance and failure modes, with implications for clinical orthodontic practice.

It is essential to acknowledge that *in vitro* studies have inherent limitations when attempting to replicate the complex oral environment accurately. The distinction in force application points could potentially influence the results by introducing varied stress distributions on the bracket base. Altering the location of force application might lead to differences in SBS values and failure modes, consequently impacting the overall study outcomes. The distance between the force application point and the enamel surface plays a crucial role in determining the stress experienced by the adhesive interface, potentially affecting the bond strength results. Therefore, varying the points of force application on the bracket base could result in diverse outcomes, emphasising the significance of considering the location of force application in orthodontic bond strength testing methodologies [23, 24].

Also, factors including pH and temperature variations, stresses produced from an activated arch wire combined with occlusal forces, and the presence of complex oral microflora cannot be replicated in a simulated testing environment [9]. Laboratory bond strength studies are not a substitute for *in vivo* testing; however, they provide us with a preliminary understanding of how materials may perform in the oral environment [1, 20].

## Conclusion

Based on this *in vitro* study, the following conclusions could be drawn:

1. The bracket base shape has an effect on SBS. The rectangular (control) base shape had a higher mean SBS compared to flower and heart base shapes.
2. All bracket shapes had bond strengths above the clinically acceptable range of 6–8 MPa, and may potentially provide adequate SBS values in a clinical situation.
3. The SBS values for shaped bracket bases do not change significantly between 24 h and 2 months.
4. Variations in ARI scores also occur based on bracket shape.
